# A year living with SARS-CoV-2: an epidemiological overview of viral lineage circulation by whole-genome sequencing in Barcelona city (Catalonia, Spain)

**DOI:** 10.1080/22221751.2021.2011617

**Published:** 2022-01-04

**Authors:** Cristina Andrés, Maria Piñana, Blanca Borràs-Bermejo, Alejandra González-Sánchez, Damir García-Cehic, Juliana Esperalba, Ariadna Rando, Ricardo-Gabriel Zules-Oña, Carolina Campos, Maria Gema Codina, Albert Blanco-Grau, Sergi Colomer-Castell, Maria Carmen Martín, Carla Castillo, Karen García-Comuñas, Rodrigo Vásquez-Mercado, Reginaldo Martins-Martins, Narcís Saubi, Magda Campins-Martí, Tomàs Pumarola, Josep Quer, Andrés Antón

**Affiliations:** a Respiratory Viruses Unit, Microbiology Department, Vall d’Hebron Institut de Recerca (VHIR), Vall d’Hebron Barcelona Hospital Campus Barcelona, Spain; b Preventive Medicine and Epidemiology Department, Vall d´Hebron Research Institute (VHIR), Vall d’Hebron Barcelona Hospital Campus, Barcelona, Spain; c Liver Diseases-Viral Hepatitis, Liver Unit, Vall d’Hebron Institut de Recerca (VHIR), Vall d’Hebron Barcelona Hospital Campus, Barcelona, Spain; d Centro de Investigación Biomédica en Red de Enfermedades Hepáticas y Digestivas (CIBERehd), Instituto de Salud Carlos III, Madrid, Spain; e Clinical Biochemistry (Clinical Laboratories), Vall d'Hebron Barcelona Hospital Campus, Barcelona, Spain; f Respiratory Viruses Unit, Microbiology Department, Vall d'Hebron Institut de Recerca (VHIR), Vall d'Hebron Barcelona Hospital Campus, Passeig Vall d'Hebron, Barcelona, Spain

**Keywords:** SARS-CoV-2, COVID-19, molecular epidemiology, genetic diversity, Catalonia

## Abstract

Herein, we describe the genetic diversity of circulating SARS-CoV-2 viruses by whole-genome sequencing (WGS) in Barcelona city (Catalonia, Spain) throughout the first four pandemic waves. From weeks 11/2020–24/2021, SARS-CoV-2-positive respiratory samples were randomly selected per clinical setting (80% from primary care or 20% from the hospital), age group, and week. WGS was performed following the ARTICv3 protocol on MiSeq or NextSeq2000 Illumina platforms. Nearly complete consensus sequences were used for genetic characterization based on GISAID and PANGOLIN nomenclatures. From 2475 samples, 2166 (87%) were fully sequenced (78% from primary care and 22% from hospital settings). Multiple genetic lineages were co-circulating, but four were predominant at different periods. While B.1.5 (50.68%) and B.1.1 (32.88%) were the major lineages during the first pandemic wave, B.1.177 (66.85%) and B.1.1.7 (83.80%) were predominant during the second, third, and fourth waves, respectively. Almost all (96.4%) were carrying D614G mutation in the S protein, with additional mutations that define lineages or variants. But some mutations of concern, such as E484K from B.1.351 and P.1 lineages are currently under monitoring, together with those observed in the receptor-binding domain or N-terminal domain, such as L452R and T478K from B.1.617.2 lineage. The fact that a predominant lineage was observed in each pandemic wave suggests advantageous properties over other contemporary co-circulating variants. This genetic variability should be monitored, especially when a massive vaccination campaign is ongoing because the potential selection and emergence of novel antigenic SARS-CoV-2 strains related to immunological escapement events.

## Introduction

An outbreak of coronavirus disease (COVID-19), caused by severe acute respiratory syndrome coronavirus 2 (SARS-CoV-2), emerged in Wuhan (Hubei Province, China) in late December 2019. After spreading worldwide, a global pandemic was finally declared by the World Health Organisation (WHO) on 11 March [1,2].

SARS-CoV-2 virus belongs to the *Coronaviridae* family, within the beta-coronavirus genera, together with other human seasonal (hCoV-OC43 or hCoV-HKU) and potential pandemic (SARS-CoV or Middle East Respiratory Syndrome (MERS)-CoV) coronaviruses [3,4]. It is an enveloped, single-stranded and positive-sensed RNA virus whose genome (about 30 kb) encodes for 4 major structural proteins (Spike, S; Envelope, E; Membrane, M; and Nucleocapsid, N), 16 non-structural proteins (nsp1-16) encoded by the open reading frame (ORF) 1ab, and six accessory proteins (ORF 3a, 6, 7a, 7b, 8, and 10) [1]. According to the SARS-CoV-2 submitted sequences to GISAID (https://www.gisaid.org; 21 July 2021), up to 9 clades and subclades (O, S, L, V, G, GV, GH, GR, GRY) have been distinguished worldwide, which included numerous lineages A and B, according to Pangolin nomenclature (https://cov-lineages.org/) [5–7].

SARS-CoV-2 has been a cause of an important health burden in Spain. The first case was laboratory-confirmed in Spain at the end of January 2020. Afterwards, a National lockdown was imposed from 14 March to 4 May to control viral dissemination during the first pandemic wave [8], in which schools remained closed and teleworking prevailed [9,10]. Progressive de-escalation of restrictive measures occurred until 21 June 2020, when the population got back to normal activities applying recommended non-pharmaceutical interventions as hand washing, wearing a face mask, and social distancing. Moreover, primary healthcare centres (first level of contact with healthcare system for community) strengthened their testing capacity and contact tracing activities, playing a key role for prompt microbiological confirmation and control the pandemic. After that, up to three subsequent pandemic waves (second, from epidemiological week 27/2020 to 52/2020; third, from 53/2020 to 12/2021; and fourth, from 13/2021 to 24/2021) were reported later peaking in October 2020, January 2021, and April 2021, respectively. Herein, we describe the molecular epidemiology by whole-genome sequencing (WGS) of circulating SARS-CoV-2 strains detected at a tertiary university hospital and primary care settings in Barcelona city beyond the first pandemic year, until June 2021.

## Material and methods

### Patients and samples

Upper respiratory tract specimens (nasopharyngeal aspirates and naso/oropharyngeal swabs) were collected for SARS-CoV-2 laboratory confirmation from patients accomplishing the case definition criteria of SARS infection [11] attended at the emergency department, admitted to the Hospital Universitari Vall d’Hebron (HUVH) or at primary care centres in Barcelona. Up to 99 primary care centres, comprising all the Barcelona metropolitan area inhabitants, have our hospital as the reference site for laboratory confirmation of SARS-CoV-2 suspected cases. Demographic features (sex and age) were collected from SARS-CoV-2 laboratory-confirmed cases. From epidemiological week 11/2020 (March 2020) to epidemiological week 24/2021 (June 2021), samples were randomly selected for WGS from laboratory-confirmed cases according to the following criteria: equally per sex, per age group (0–4, 5–14, 15–40, 41–64, and >64 years) and per origin centre (hospital or primary care), when possible. Only first respiratory samples per patient were included. Between 1 and 5% of laboratory-confirmed cases were selected depending on the SARS-CoV-2 laboratory-confirmed detected each week.

### Detection of SARS-CoV-2 in respiratory specimens

Several methods were used for SARS-CoV-2 detection throughout the study period. Detection of SARS-CoV-2 was firstly performed by an in-house PCR assay with primers and probes from 2019-nCoV CDC PCR panel and using the One-Step RT–PCR kit (Qiagen, Germany). When commercial assays became available, real-time multiplex RT–PCR assays like Allplex™ 2019-nCoV Assay (Seegene, South Korea) and Cobas® SARS-CoV-2 Test (Roche Diagnostics, USA) were used, which were replaced by other high-throughput automated transcription-mediated amplification based-assays (Procleix SARS-CoV-2, Grifols, Spain; Aptima SARS-CoV-2, Hologic Inc., USA) on Panther platforms due to the high demand from the hospital and primary care settings.

### WGS of SARS-CoV-2

WGS of SARS-CoV-2 from selected specimens was performed following the ARTIC Network protocol (https://artic.network/ncov-2019). Briefly, cDNA synthesis was performed with SuperScript IV reverse transcriptase (Invitrogen, USA) and further full-genome amplification with ARTIC V3 primer sets (Integrated DNA Technologies, IDT, USA), with Q5 Hot Start High-Fidelity DNA polymerase (New England BioLabs, USA). Library preparation was performed using the KAPA HyperPrep Kit (Roche Applied Science, USA) or Illumina DNA Prep (Illumina, USA). All samples were finally normalized to 4 nM, pooled together with a 5% of PhiX internal DNA control (*PhiX V3*, Illumina, USA), and loaded in a MiSeq or Nextseq 2000 P2 Reagent Kits 600v3 and 200v3 cartridges (Illumina, USA), respectively.

### Bioinformatic analysis of raw fastQ files

Two fastQ files were generated for each patient (Read 1, R1; and, Read 2, R2) after the sequencing procedure. All data were uploaded to BaseSpace Sequence Hub (Illumina, USA) to perform the mapping to the SARS-CoV-2 reference genome (Wuhan; NC_045512.2) and to report the genome coverage and sequencing depth using the DRAGEN COVID Lineage App (v3.5.2, Illumina, USA). This App performs Kmer-based detection followed by Map/Align, Variant Calling, and Consensus Sequence generation. Furthermore, it performs lineage/clade determination and mutation characterization by using updated Pangolin (https://cov-lineages.org/pangolin.html) and NextClade (https://clades.nextstrain.org/) nomenclatures. Only those sequences with good quality (>80% genome coverage and minimum depth of 100X) [12] were used for molecular characterization of the S protein in comparison with the reference genome (NC_045512.2), using MEGA v6 [13], and also uploaded to GISAID database [14]. Additionally, the evolutionary divergence within and between genetic groups of whole-genome and *Spike* sequences depending on the pandemic wave was performed with *p*-distance method in MEGA v6 [13].

## Results

**Figure 1.**
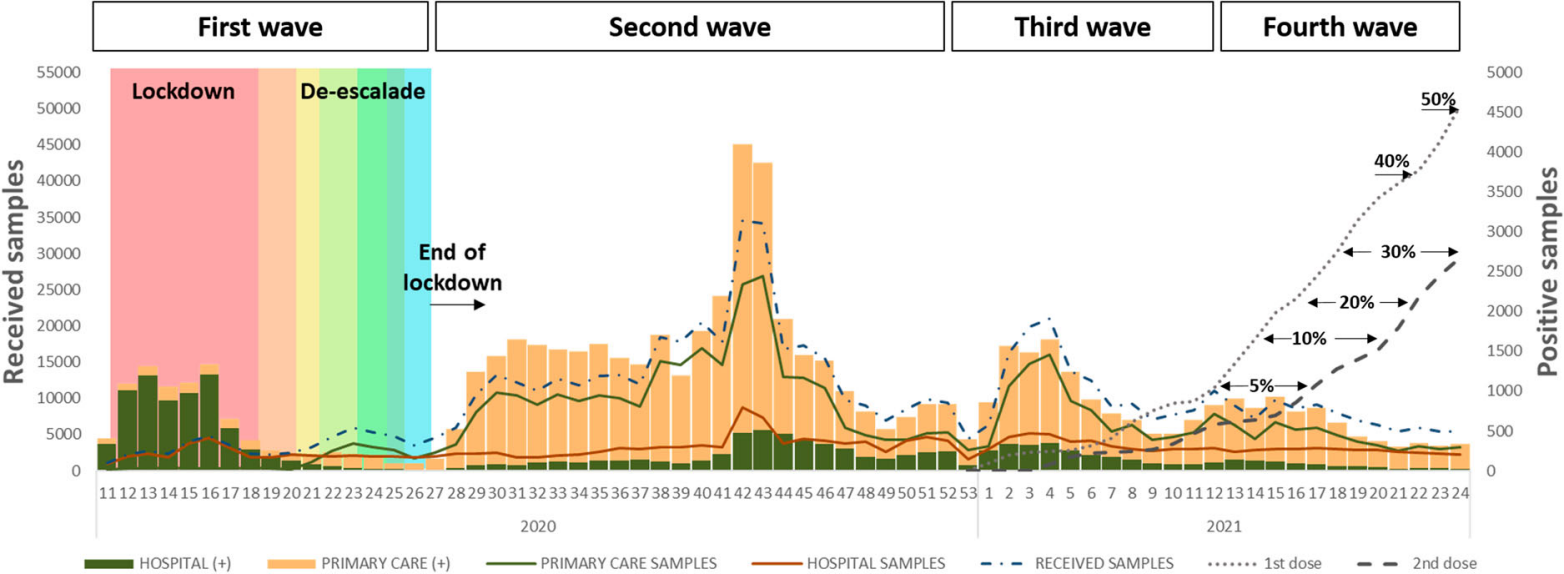
Weekly distribution of received and SARS-CoV-2 laboratory-confirmed specimens from Hospitals (green) and Primary Care Centres (brown). Lockdown and the different de-escalation phases are labelled in colours. Vaccine coverages from people living in Barcelona are represented by dot line (for one dose) and dash line (for two doses).

From week 11 (March 2020) to week 24 (June 2021), 655,760 samples were received for SARS-CoV-2 laboratory confirmation, 207,109 (32%) from HUVH, and 448,651 (68%) from primary care setting, of which 65,616 (10%) samples were positive, 15,303 (23%) from HUVH, and 50,313 (77%) from primary care. Demographic data from all and positive cases and the community vaccination coverages (https://dadescovid.cat) are summarized by pandemic waves in Table 1 and Figure 1. At the beginning of the first wave (weeks 11–18/2020), most tested samples were collected from hospitalized or attended patients (20,521; 88%), but progressively the percentage of samples received from primary care increased (weeks 19–26: 50 vs 50%) but showing a lower SARS-CoV-2 positivity rate than those from the hospital setting. During the following waves, instead, most samples came from primary care (second wave: 75 vs 25%; third wave: 69 vs 31%; and fourth wave: 62 vs 38%) as described in Table 1. Moreover, the age distribution of the tested population during the different pandemic waves changed throughout the study period, and while the >64 age group was the most represented at the beginning (first wave), younger population (15–40 years) was more reported later. As the percentage of vaccinated population has been raising during the last months, and the youngest being the last prioritized cohort for vaccination, the median age of infected patients from primary care was younger wave after wave, while in the hospital, more than a half of the confirmed patients were >50 years. It is worth highlighting the low positivity among paediatric population (<14 years) in hospital (range from 8 to 4%) and primary care (range from 4 to 28%).

**Table 1.** Distribution of received and SARS-CoV-2 laboratory-confirmed specimens per origin, age group, and sex in each pandemic wave.

	1st wave (weeks 11–26)								2nd wave (weeks 27–52)							
Total/positive (%)	55,625 / 8755 (15.7%)								366,511 / 38,142 (10.4%)							
Origin	HUVH				Primary care				HUVH				Primary care			
	Tested		Positive		Tested		Positive		Tested		Positive		Tested		Positive	
Total (%)/positive (%)*	36,734	66%	6972	19%	18,891	44%	1783	9%	91,298	25%	4996	5%	27,5213	75%	33,146	12%
**Median age (IQR)**	**55 (38–75)**	**58 (42–79)**	**49 (35–66)**	**60 (44–82)**	**48 (30–67)**	**51 (33–72)**	**32 (16–51)**	**38 (22–55)**	** **	** **	** **	** **	** **	** **	** **	** **
Age group (years)**	**Total**	**%**	**+**	**%**	**Total**	**%**	**+**	**%**	**Total**	**%**	**+**	**%**	**Total**	**%**	**+**	**%**
**<5**	1133	3%	52	5%	430	2%	6	1%	4627	5%	109	2%	15,130	6%	943	6%
**5–14**	841	2%	23	3%	467	3%	13	3%	3819	4%	96	3%	44,087	16%	3007	7%
**15–40**	8276	23%	1430	17%	5723	30%	395	7%	29030	32%	1758	6%	111,117	40%	15,049	14%
**41–64**	12,244	33%	2441	20%	7368	39%	614	8%	29305	32%	1591	5%	72,120	26%	10,170	14%
**> 64**	14,240	39%	3026	21%	4903	26%	755	15%	24517	27%	1442	6%	32,759	12%	3977	12%
**% female**	21,167	58%	3985	19%	11,965	63%	1166	10%	52074	57%	2672	5%	157,399	57%	17,957	11%
**% male**	15,351	42%	2964	19%	6924	37%	616	9%	39094	43%	2321	6%	117,769	43%	15,178	13%

3rd wave (weeks 53–12)								4th wave (weeks 13–24)							
146,504 / 11,866 (8.1%)								87,120 / 6853 (7.9%)							
**HUVH**				**Primary care**				**HUVH**				**Primary care**			
**Tested**		**Positive**		**Tested**		**Positive**		**Tested**		**Positive**		**Tested**		**Positive**	
45,927	31%	2475	5%	100,577	69%	9391	9%	33,150	38%	860	3%	53,970	62%	5993	11%
51 (32–70)	58 (35–76)	35 (16–53)	38 (20–55)	55 (32–72)	60 (39–76)	27 (15–45)	33 (17–49)								
Total	%	+	%	Total	%	+	%	Total	%	+	%	Total	%	+	%
1804	4%	32	2%	1882	2%	295	16%	2396	7%	21	1%	1503	3%	272	18%
4597	10%	187	4%	7285	7%	845	12%	11,715	35%	369	3%	6053	11%	600	10%
13,690	30%	680	5%	36,630	36%	3499	10%	7826	24%	194	2%	24,359	45%	2475	10%
14,360	31%	729	5%	39,902	40%	3286	8%	9778	30%	254	3%	17,328	32%	1838	11%
11,476	25%	847	7%	14,878	15%	1466	10%	1435	4%	22	2%	4727	9%	808	17%
26,384	57%	1384	5%	68,246	68%	5502	7%	17,544	53%	458	3%	30,727	57%	3135	10%
19,537	43%	1091	6%	32,307	32%	3888	9%	15,603	47%	402	3%	23,242	43%	2858	12%

*Percentages of total samples received and positive by pandemic wave were calculated relative to the total specimens shown in the second row; percentages from positive samples were calculated depending on the total specimens received per pandemic wave and site.

**Percentages of total samples received by age group were calculated relative to the total specimens by clinical setting and pandemic wave shown in the fourth row; percentages from positive samples were performed horizontally, depending on the total specimens received per age group

**Figure 2.**
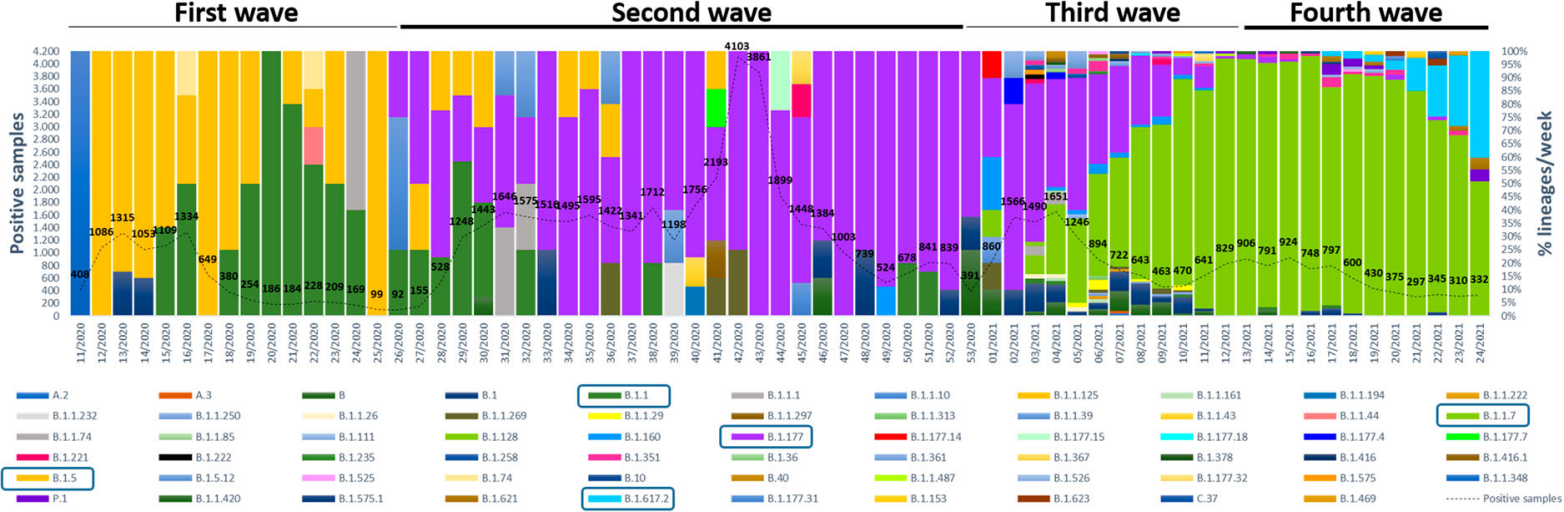
Weekly distribution of the different lineages during the study period (weeks 11/2020–24/2021) labelled in colours. Dashed line represents all SARS-CoV-2 laboratory-confirmed samples. Those prevalent lineages in each pandemic wave are blue squared.

A total of 2468 (4%) SARS-CoV-2 laboratory-confirmed samples were weekly selected according to the criteria listed above, 541 (22%) from hospital setting and 1927 (78%) from primary care. The genetic characterization of 2166 (88%) viruses by successful WGS revealed the absolute circulation of viruses belonging to lineage B since March 2020, but three cases to lineage A. As represented in Table 2 and in Figure 2, most viruses fell into lineages B.1.1.7 (1353/2166; 62.47%), B.1.177 (367/2166; 16.94%), B.1.617.2 (71/2166; 3.28%), B.1 (57/2166; 2.63%), B.1.5 (49/2166; 2.26%) and B.1.1 (48/2166; 2.22%), in addition to other minor lineages, which were predominant at different periods. At the beginning of the first wave, B.1.5 viruses (50.68%) dominated in the hospital setting until week 19/2020, when cases related to B.1.1 viruses (32.88%) significantly increased together with the emergence of other minor lineages, such as B.1.74 or B.1.1.44, coinciding with the start of de-escalation and the strengthen of primary care testing capacity (Figure 2). Since week 26/2020, when first B.1.177 cases were detected, its prevalence increased along the whole summer until the end of the year, becoming the predominant lineage during the second wave. Just the beginning of 2021 was characterized by the introduction and spread of variant B.1.1.7 (variant Alpha), which progressively replaced B.1.177 as the dominant lineage during the late third (55.44%) and early fourth (85.80%) waves, when became and remained predominant. Similarly, B.1.617.2 (variant Delta)-related cases raised (40%), becoming the predominant lineage weeks later the end of the study.

**Table 2.** Lineage observation overall, and per pandemic wave during the study period (% per pandemic wave).

	Global		1st wave		2nd wave		3rd wave		4th wave	
	Lineage	*N* (%)	Lineage	*N* (%)	Lineage	*N* (%)	Lineage	*N* (%)	Lineage	*N* (%)
**B**	**B.1.1.7**	**1353** (**62.47)**			** **	** **	**B.1.1.7**	**525** (**55.44)**	**B.1.1.7**	**828** (**85.80)**
** **	**B.1.177**	**367** (**16.94)**	B.1.177	1 (1.37)	**B.1.177**	**121** (**66.85)**	**B.1.177**	**238** (**25.13)**	B.1.177	7 (0.73)
** **	**B.1.617.2**	**71** (**3.28)**	** **	** **			** **	** **	**B.1.617.2**	**71** (**7.36)**
** **	**B.1**	**57** (**2.63)**	B.1	2 (2.74)	B.1	5 (2.76)	B.1	43 (4.54)	B.1	7 (0.73)
** **	**B.1.5**	**49** (**2.26)**	**B.1.5**	**37** (**50.68)**	B.1.5	12 (6.63)				
** **	**B.1.1**	**48** (**2.22)**	**B.1.1**	**24** (**32.88)**	B.1.1	19 (10.50)	B.1.1	3 (0.32)	B.1.1	2 (0.21)
** **	B	35 (1.62)			B	2 (1.10)	B	31 (3.27)	B	2 (0.21)
** **	B.1.351	19 (0.88)					B.1.351	7 (0.74)	B.1.351	12 (1.24)
** **	B.1.160	17 (0.78)			B.1.160	1 (0.55)	B.1.160	16 (1.69)	** **	** **
** **	P.1	14 (0.65)					P.1	2 (0.21)	P.1	12 (1.24)
** **	B.1.361	13 (0.60)					B.1.361	13 (1.37)		
** **	B.1.1.269	9 (0.42)			B.1.1.269	3 (1.65)	B.1.1.269	6 (0.63)	** **	** **
** **	B.1.621	7 (0.32)							B.1.621	7 (0.73)
** **	B.1.1.29	7 (0.32)					B.1.1.29	7 (0.74)		
** **	B.1.1.1	6 (0.28)	B.1.1.1	3 (4.11)	B.1.1.1	3 (1.65)			** **	** **
** **	B.1.1.74	5 (0.23)					B.1.1.74	5 (0.53)		
** **	B.1.1.250	4 (0.18)					B.1.1.250	4 (0.42)		
** **	B.1.221	4 (0.18)			B.1.221	1 (0.55)	B.1.221	3 (0.32)	** **	** **
** **	B.1.526	4 (0.18)					B.1.526	1 (0.11)	B.1.526	3 (0.31)
** **	B.1.416.1	4 (0.18)					B.1.416.1	4 (0.42)		
** **	B.1.1.26	3 (0.14)					B.1.1.26	3 (0.32)	** **	** **
** **	B.1.623	3 (0.14)			B.1.1.39	1 (0.55)			B.1.623	3 (0.31)
** **	B.1.5.12	3 (0.14)			B.1.5.12	3 (1.65)				
** **	B.1.177.32	3 (0.14)					B.1.177.32	3 (0.32)	** **	** **
** **	B.1.1.10	3 (0.14)	B.1.1.10	2 (2.74)	B.1.1.10	1 (0.55)				
** **	B.1.177.4	3 (0.14)					B.1.177.4	3 (0.32)	** **	** **
** **	C.37	2 (0.09)							C.37	2 (0.21)
** **	B.1.1.222	2 (0.09)					B.1.1.222	2 (0.21)		
** **	B.40	2 (0.09)					B.40	2 (0.21)	** **	** **
** **	B.1.177.15	2 (0.09)			B.1.177.15	2 (1.10)				
** **	B.1.575.1	2 (0.09)							B.1.575.1	2 (0.21)
** **	B.1.74	2 (0.09)	B.1.74	2 (2.74)					** **	** **
** **	B.1.1.161	2 (0.09)					B.1.1.161	2 (0.21)		
** **	B.1.1.487	2 (0.09)					B.1.1.487	2 (0.21)		
** **	B.1.235	2 (0.09)					B.1.235	2 (0.21)	** **	** **
** **	B.1.1.420	2 (0.09)							B.1.1.420	2 (0.21)
** **	B.1.258	2 (0.09)					B.1.258	2 (0.21)		
** **	B.1.153	2 (0.09)							B.1.153	2 (0.21)
** **	B.1.36	2 (0.09)					B.1.36	2 (0.21)		
** **	B.1.525	2 (0.09)					B.1.525	1 (0.11)	B.1.525	1 (0.10)
** **	B.1.1.39	2 (0.09)					B.1.1.39	1 (0.11)	** **	** **
** **	B.1.1.297	1 (0.05)			B.1.1.297	1 (0.55)				
** **	B.1.575	1 (0.05)					B.1.575	1 (0.11)		
** **	B.1.367	1 (0.05)			B.1.367	1 (0.55)			** **	** **
** **	B.1.1.313	1 (0.05)					B.1.1.313	1 (0.11)		
** **	B.1.1.232	1 (0.05)			B.1.1.232	1 (0.55)				
** **	B.1.111	1 (0.05)					B.1.111	1 (0.11)	** **	** **
** **	B.1.1.43	1 (0.05)			B.1.1.43	1 (0.55)				
** **	B.1.378	1 (0.05)					B.1.378	1 (0.11)	** **	** **
** **	B.1.1.125	1 (0.05)			B.1.1.125	1 (0.55)				
** **	B.1.1.348	1 (0.05)					B.1.1.348	1 (0.11)		
** **	B.1.222	1 (0.05)					B.1.222	1 (0.11)	** **	** **
** **	B.1.177.14	1 (0.05)					B.1.177.14	1 (0.11)		
** **	B.10	1 (0.05)					B.10	1 (0.11)		
** **	B.1.1.85	1 (0.05)					B.1.1.85	1 (0.11)		
** **	B.1.177.18	1 (0.05)					B.1.177.18	1 (0.11)		
** **	B.1.177.31	1 (0.05)							B.1.177.31	1 (0.10)
** **	B.1.469	1 (0.05)							B.1.469	1 (0.10)
** **	B.1.128	1 (0.05)					B.1.128	1 (0.11)		
** **	B.1.1.44	1 (0.05)	B.1.1.44	1 (1.37)						
** **	B.1.416	1 (0.05)					B.1.416	1 (0.11)		
** **	B.1.1.194	1 (0.05)			B.1.1.194	1 (0.55)				
** **	B.1.177.7	1 (0.05)			B.1.177.7	1 (0.55)				
**A**	A.2	2 (0.09)	A.2	1 (1.37)			A.2	1 (0.11)		
** **	A.3	1 (0.05)					A.3	1 (0.11)		
	**Total**	**2166**	**Total**	**73** (**3.4%)**	**Total**	**181** (**8.4%)**	**Total**	**947** (**43.7%)**	**Total**	**965** (**44.5%)**

On the other hand, the evolutionary divergences observed throughout the whole viral genome and *Spike* sequences within and between group of sequences for each wave are described in Table 3. Differences in genetic intragroup divergences were observed in the whole genomes per waves, which tripled from the first to third and fourth waves, especially higher in the *Spike* gene sequence, from 0.00017 (first wave) to 0.00136 (third wave) and 0.00097 (fourth wave). Intergroup divergence comparison revealed a higher diversification, from 0.0004 (first wave) to 0.0013 (third wave) and 0.0018 (fourth wave).

**Table 3.** Evolutionary divergences on whole viral (29764 nt) and *Spike* (3822 nt) sequences within and between waves.

Whole genome					Spike				
Within waves									
Waves	1st	2nd	3rd	4th	Waves	1st	2nd	3rd	4th
	0.00027	0.00054	0.00100	0.00086		0.00017	0.00044	0.00136	0.00097
**Between waves**									
**Waves**	**1st**	**2nd**	**3rd**	**4th**	**Waves**	**1st**	**2nd**	**3rd**	**4th**
**1**	-	-	-	-	**1**	-	-	-	-
**2**	0.0005	-	-	-	**2**	0.0004	-	-	-
**3**	0.0009	0.0010	-	-	**3**	0.0012	0.0014	-	-
**4**	0.0012	0.0014	0.0010	-	**4**	0.0018	0.0021	0.0014	-

When the consensus S-coding sequences of 1921 (90%) SARS-CoV-2 viruses were compared (Supplementary Table 1), a total of 276 amino acid substitutions were found. Most changes were observed in the N-terminal domain (NTD) (92/276; 33%) and the receptor-binding domain (RBD) (40/276; 14%). The D614G (96.4%) substitution was observed in most viral genomes, in addition to multiple mutations defining lineages, such as the mutation set Δ69–70, Δ144, N501Y, A570D, P681H, T716I, S982A, and D1118H for B.1.1.7 viruses. Other mutations of concern such as E484K, usually observed in B.1.351, B.1.525, B.1.621, and P.1 lineages are currently being monitored as well as the gain of changes in the RBD (L452R and T478 K) reported on B.1.617.2 viruses.

## Discussion

SARS-CoV-2-related outbreak was declared by the WHO as a public health emergency of international concern on 30 January 2020 due to an increase in the number of imported cases worldwide [15]. First cases in Catalonia were detected by the end of February 2020, and WGS was implemented to weekly monitor the genetic diversity and features of circulating viruses in Barcelona (Catalonia, Spain), firstly in the hospitalized population and later in the community.

During the first wave, most received samples came from hospitalized, as SARS-CoV-2 testing was only available for patients at high risk of severe disease. At the end of this wave, testing capacity was strengthened in primary care settings to confirm primary cases and engage contact tracing. Therefore, during the following waves, the percentage of samples received from primary care was higher than hospitalized patients, even when rapid antigen testing was already implemented in primary care to shorten the result turn-around time and the pressure to clinical laboratories. This measure was crucial for the correct management of COVID-19 pandemic, after these first challenging weeks, since the strengthening of primary care let to promptly detect new COVID-19 cases, reduce hospital burden and assess the compliance of control measures [16].

As shown in Table 1, the age group distribution of SARS-CoV-2 laboratory-confirmed cases was different between the four consecutive pandemic waves, in which shifted from elderly (>64 years) during the first wave to younger people (15–64 years) in the following waves. Spanish seroprevalence studies performed at the end of lockdown demonstrated that disease incidence was equally distributed across all age groups [17]. However, our results based on data mostly from the hospitalized population during the first wave, reflected that clinical burden was especially higher in the elderly, the most vulnerable population for severe illness to COVID-19 due to a lower host humoral immune response (inmunosenescence), or the existence of comorbidities [18]. Nevertheless, the progressive increase percentage of positive samples in the younger population could be explained by several reasons. First, by the higher availability of testing capacities in primary care starting in May–June 2020, revealing the equally disease distribution above mentioned that was underestimated until that moment by limited testing capacity. Second, preventive and control measures were strictly implemented in nursing homes, lowering the infection rate of the most affected population during the first wave, together with increasing social interactions among young people during the de-escalation phases. Third, a vaccination campaign against COVID-19 starting in late December 2020 prioritized the elderly population, and progressively advanced towards earlier ages, increasing the relative incidence rate in the youngest due to the low vaccination coverage, as observed in the study period [19–21].

Regarding lineage circulation, although lineages A and B have co-circulated since January 2020 at variable prevalence, temporal and geographical distributions [5–7], a greater burden and genetic heterogeneity of lineage B have been described [22]. An early diversification of B sub-lineages was detected during the early first wave, that later evolved in numerous related lineages such as those reported in this work and elsewhere [5,23]. The weekly distribution of SARS-CoV-2 viruses characterized by WGS allowed the observation of a variable predominance of a few among multiple lineages, with the emergence and extinction of these during the four pandemic waves. Overall, the most prevalent lineage detected during the study was B.1.1.7 due to its long-lasting circulation during the third and fourth waves as observed in other European countries [24,25], followed by the circulation of B.1.177 during the second wave, as reported worldwide [26–28]. However, the highest incidence reported for B.1.1.7 viruses was also due to the reinforced capability of sequencing for SARS-CoV-2 surveillance since December 2020 in our country to monitor the spread of this variant of concern.

In March 2020, a 6-week lockdown was imposed in our country as in other European regions [9]. Initially, lineage B.1.5 was the most predominant among our series, as observed in other Spanish and European regions [23,29], until the emergence and spread of B.1.1, as well as other minor lineages. The increase in B.1.1 coincided not only with the start of de-escalation phases on the basis of our government recommendations [10], but also with the beginning of diagnosis at primary care corresponding to the community living in Barcelona. Most circulating B-viruses were carrying the amino acid substitution D614G in the Spike, which appeared by the end of March 2020 and is present in most later circulating lineages belonging to GISAID’s G clade [14,22]. This major predominance of variants carrying this mutation was because D614G improves viral infectivity and viral transmission giving an advantage to virus by relaxing the trimeric Spike structure and facilitating the viral entry to the cell, as previously reported [5,30,31].

Moreover, the co-circulation of B.1.1 together with other sub-lineages was also reported in many countries [23,29,32], until the start of summer holidays (July 2020), in which B.1.177 was first detected and spread during the whole second wave, and at the time in which Spain had the lowest transmission rates (https://cnecovid.isciii.es/covid19/#ccaa). Lineage B.1.177 is supposed to have emerged from an outbreak occurred in the community of temporary fruit workers in Aragon and Catalonia (province of Lleida), and rapidly disseminated through the country and close European areas [26] as a result of opening borders in summer 2020 becoming the major lineage observed during the second half of 2020 (second wave), not only in Spain, but also in European countries like the United Kingdom, Ireland, Denmark, Italy [26], and even in Canada [33]. Epidemiological data also showed that at the time B.1.177 appeared, the competition with other lineages was very low since incidence rates were close to zero here in Spain as a consequence of the strict lockdown imposed in our country. Nonetheless, B.1.177 had the opportunity to disseminate during de-escalation and summer time [6,26]. The fact that B.1.177 was defined by the acquisition of the A222V mutation in the Spike could also confer a significant advantage to the virus, favouring its rapid selection and dissemination. Furthermore, this lineage co-circulated alongside with other minor sub-lineages (B.1.160, B.1.1, or B.1.1.10) in our region, but predominant in different European regions, such as B.1.160 in France or B.1.1 in other countries [22,26].

Nevertheless, the circulation of B.1.177 ran into the emergence of B.1.1.7 at the end of 2020, and although both lineages co-circulated during the first weeks, B.1.1.7 outcompeted the other lineages progressively, especially B.1.177, by the end of the third wave. B.1.1.7 emerged at Christmas time in the United Kingdom [24], although was first detected in September 2020, characterized by multiple mutations in the Spike, and rapidly spread to other European regions later [34,35] and United States [25] during 2021. Thus, SARS-CoV-2 lineages that circulated in our country were similar to the described elsewhere, revealing the uniform global distribution in European countries after releasing of mobility restrictions. In the United Kingdom, the emerging B.1.1.7 caused the major hospital burden during the pandemic in this country, and greater transmissibility and severity were attributed to this variant [36]. However, we could not relate any specific lineage with higher severity throughout the study period (understood as a higher positive proportion from hospitalized patients) [37]. In fact, the introduction and predominance of B.1.1.7 in Spain did not trigger a fourth wave with a high hospital burden, but it probably contributed to maintain a high community transmission rates due to its enhanced transmissibility and the increased social interactions between younger adults after lockdown [35,38]. Differently from the second wave with B.1.177 viruses, B.1.1.7 replaced all lineage competitors, until the upsurge of B.1.617.2, that reached close to 40% prevalence by mid-June 2021 driving to the fifth pandemic wave in our country [39]. Nevertheless, due to the implementation of vaccination, the incidence was skewed to the youngest group (15–40), as they remained with low vaccine coverage rates until the end of 2021 summer.

Before massive vaccination campaigns, the selective pressure under genetic evolution of SARS-CoV-2 was very low as observed in the different intra- and intergroup divergences. However, many mutations were observed in the Spike among the SARS-CoV-2 viruses in our series. Additionally to the key defining-mutations of B.1.5, B.1.1, B.1.177, and B.1.1.7-related viruses, other substitutions such as S943I associated with a reduced Spike stability [40], or A262S, also observed among mink-derived SARS-CoV-2 variants [41], have not caught special attention since they were not fixed in any predominant lineage. Moreover, differences in lineage predominance along the four pandemic waves were also concordant with the evolutionary divergences observed within and between viruses during each wave in the present study. The intragroup divergence was tripled in the third or fourth among the *Spike* sequences and intergroup divergences. All remark that last circulating variants have substantially evolved and acquired advantage mutations, especially on key regions of the Spike*,* to better promote the viral persistence. Interestingly, during the third wave, a great number of new lineages, carrying multiple mutations in the Spike, emerged (B.1.351, P.1, and B.1.1.7) [24,35]. Despite the moderate mutation rate of SARS-CoV-2 [42], selection of new variants and mutations, especially in the Spike, occurred as an adaptation mechanism to the increased environmental pressure explained by an increasing population immunity favoured by an increasing mobility.

Therefore, one of the substitutions of interest and shared by the new variants is N501Y, located at the RBD of Spike, which increases ACE2 binding affinity, and improves the human-to-human transmission [43]. The same activity is described for E484K substitution, shared by B.1.351 and P.1 lineages, and reported during the initial weeks of the third wave in our study, as in other European regions [28,35]. Nonetheless, the prevalence of E484K mutation among other lineages also increased in many countries due to its benefits for SARS-CoV-2, not only for its major transmissibility, but also evading antibody neutralization from host immune response [6,44]. Furthermore, during the fourth wave, an increasing prevalence of lineage B.1.617.2 and AY sub-lineages was observed, replacing most of all previous circulating lineages in mid-July (data not shown). This emergent variant is carrying mutations in the RBD (L452R and T478K) and in the polybasic region (P681R) [45]. The L452R mutation was previously observed in the United States due to the higher circulation of lineages B.1.427 and B.1.429 (variant Epsilon) and related to a partial immune evasion due to the disruption to the RBD binding together with T478K substitution [45–47]. Also, P681R is shared by other lineages, and it was firstly reported for lineage A.23.1 [48]. This mutation extends the polybasic motif, and it might enhance the viral replication and transmission through the higher processing by host proteases, and thus, an increased cleavability with furin [45,48]. Apart from the lineage-defining changes in variants Delta-like, additional substitutions were observed such as T95I, P251L in the N-terminal domain, Q613H and T719I during the following weeks (data not shown).

Natural viral evolution highlights the relevance of surveillance based on WGS. This virological surveillance will allow us to rapidly identify the upsurge of novel variants with phenotypic properties relevant to transmission, virulence, and immunity escapement. This is of especial relevance when vaccine coverages are high in Spain. WGS-based surveillance should be now focused on further viral characterization from breakthrough infections, reinfections, and viruses from imported cases from countries with high SARS-CoV-2 infection incidences and low vaccination coverages. Additionally, more genetic data will prospectively provide valuable information for the revision of vaccine composition, when needed.

In summary, the present study reports the molecular epidemiology of SARS-CoV-2 viruses circulating during the four pandemic waves in Barcelona, Catalonia (Spain), before high vaccine coverage rates were reached. Multiple lineages were co-circulating under a neutral selection evolution until massive vaccination campaigns started, but a different predominant lineage was observed in each pandemic wave, suggesting acquired genetic advantages over other previous circulating variants. This genetic variability must be monitored because of the likely selection of variants with novel phenotyping characteristics relevant to transmissibility, severity, or antigenic properties.
